# Claims-Based vs Agency-Reported Patient Outcomes Among Home Health Agencies, 2013-2019

**DOI:** 10.1001/jamanetworkopen.2024.5692

**Published:** 2024-04-10

**Authors:** Amanda C. Chen, Christina Xiang Fu, David C. Grabowski

**Affiliations:** 1 Department of Health Care Policy, Harvard Medical School, Boston, Massachusetts; 2 Harvard Graduate School of Arts and Sciences, Boston, Massachusetts

## Abstract

**Importance:**

Given the growth of home health agency (HHA) care, it is important to understand whether quality reporting programs, such as star ratings, are associated with improved patient outcomes.

**Objective:**

To assess the immediate and long-term association of the introduction of HHA star ratings with patient-level quality outcomes, comparing claims-based and agency-reported measures.

**Design, Setting, and Participants:**

This cross-sectional study used Medicare HHA claims and agency-reported assessments to identify sequential patient episodes (ie, spells) among US adults with traditional Medicare who received HHA care (2013-2019). An interrupted time series (ITS) model was used to measure changes in trends and levels before and after the introduction of star ratings. Statistical analysis was performed from November 2022 to September 2023.

**Exposure:**

The exposure was the introduction of HHA star ratings. The postexposure period was set as starting January 1, 2016, to account for the period when both star ratings (quality of patient care and patient satisfaction rating) were publicly reported.

**Main Outcomes and Measures:**

The main outcomes included claims-based hospitalization measures (both during the patient spell and 30 days after HHA discharge) and agency-reported functional measures, such as improvement in ambulation, bathing, and bed transferring. There was also a measure to capture timely initiation of care among post–acute care HHA users, defined as HHA care initiated within 2 days of inpatient discharge.

**Results:**

This study identified 22 958 847 patient spells to compare annual changes over time; 9 750 689 patient spells were included during the pre–star ratings period from January 1, 2013, to December 31, 2015 (6 067 113 [62.2%] female; 1 100 145 [11.3%] Black, 512 487 [5.3%] Hispanic, 7 845 197 [80.5%] White; 2 656 124 [27.2%] dual eligible; mean [SD] patient spell duration, 70.9 [124.9] days; mean [SD] age, 77.4 [12.0] years); 13 208 158 patient spells were included during the post–star ratings period from January 1, 2016, to December 31, 2019 (8 104 69 [61.4%] female; 1 385 180 [10.5%] Black, 675 536 [5.1%] Hispanic, 10 664 239 [80.7%] White; 3 318 113 [25.1%] dual eligible; mean [SD] patient spell duration, 65.3 [96.2] days; mean [SD] age, 77.7 [11.6] years). Results from the ITS models found that the introduction of star ratings was associated with an acceleration in the mean [SE] hospitalization rate during the spell (0.39% [0.05%] per year) alongside functional improvements in ambulation (2.40% [0.29%] per year), bed transferring (3.95% [0.48%] per year) and bathing (2.34% [0.19%] per year) (*P* < .001). This occurred alongside a 1.21% (0.12%) per year reduction in timely initiation of care (*P* < .001).

**Conclusions and Relevance:**

This cross-sectional study found an observed improvement in agency-reported functional measures, which contrasted with slower increases in more objective measures such as hospitalization rates and declines in timely initiation of care. These findings suggest a complex picture of HHA quality of care after the introduction of star ratings.

## Key Points

**Question:**

How have patient-level outcomes changed after the introduction of home health agency (HHA) star ratings, and do these changes vary between claims-based and HHA-reported measures?

**Findings:**

This cross-sectional study of 22 958 847 patient episodes leveraged 4 years of follow-up data after the introduction of HHA star ratings and observed higher hospitalization rates and less timely initiation of care over time. However, there was also an observed functional improvement based on HHA-reported measures during this period.

**Meaning:**

The observed improvement in HHA-reported measures was not accompanied by corresponding improvement in more objective measures (eg, hospitalizations), which raises concern about the use of agency-reported measures in assessing HHA quality of care.

## Teaser

This cross-sectional study assesses whether the introduction of home health agency star ratings was associated with patient-level quality outcomes, comparing claims-based and agency-reported measures.

## Introduction

The quality of home health agency (HHA) care has commanded increased attention in recent years, because HHAs are viewed as a lower-cost substitute for traditional institutional post–acute care (PAC) services, such as skilled nursing facilities. Currently, there are 3.1 million Medicare fee-for-service (FFS) beneficiaries who receive care from more than 11 000 HHAs.^1^ Medicare spends over $17.1 billion to cover HHA services for individuals unable to leave their homes who need part-time or intermittent skilled nursing or physical, occupational, or speech therapy services; these individuals may also receive aide and medical social work services as part of the HHA benefit.^1^ Unlike most other PAC settings, a preceding hospital stay is not needed to become eligible for services, there are no copayments or deductibles, and staff travel to a patient’s home to deliver care.^1^ As a result, ensuring transparency about the quality of care for individuals receiving HHA services has been a key initiative of the Centers of Medicare and Medicaid Services (CMS) for over 2 decades.

In fall 2003, CMS launched the Home Health Quality Initiative nationally to publicly display agency-specific quality measures.^2^ Some studies focusing on the 2003 rollout identified a small consumer response, as agencies with higher quality scores were more likely to be preferred by consumers after the initiative.^3^ Following star rating programs for other health care settings,^4^ CMS launched the HHA 5-star rating system on Care Compare to provide summary information using the number of stars to denote quality, beginning with a quality of patient care star rating in July 2015 and a patient satisfaction star rating in January 2016.^5^

Prior literature has mostly focused on the immediate effect of the introduction of star ratings and how they might be used by consumers to select care, finding small increases in the probability of selecting a high-quality HHA.^6^ These studies often examine each star rating separately, with most focused on the quality of patient care rating.^7,8^ Other work has examined factors contributing to variation in access and use of high-quality HHAs, finding that Black and Hispanic as well as lower-income HHA users have lower probabilities of high-quality HHA use compared with their White or higher income neighbors.^7,8^ It has also been noted that quality varies by profit status, chain membership, and geography.^9,10,11^

Our work complements the existing literature by providing an analysis of the longer-term association of HHA star ratings. We examine changes in patient-level outcomes prior to the introduction of the star ratings program (2013-2015) with several years of postexposure period follow-up (2016-2019) to assess differences between claims-based and HHA-reported measures.

## Methods

This cross-sectional study was deemed not to be human participants research by the Harvard Medical School institutional review board; therefore, it was exempt from further review and informed consent. We followed the Strengthening the Reporting of Observational Studies in Epidemiology (STROBE) reporting guideline.

### Data Sources

A 100% Medicare sample was obtained from the Medicare Beneficiary Summary File (MBSF),^12^ Chronic Conditions Data Warehouse (CCW),^13^ Medicare Provider Analysis and Review (MedPAR) file,^14^ Medicare FFS Outpatient file,^15^ Medicare FFS HHA claims,^16^ and the Outcome and Assessment Information Set (OASIS)^17^ for the years 2013 to 2020. All HHAs certified by Medicare are required to submit agency-reported assessments completed by HHA nurses or therapists for patients receiving skilled HHA services. We linked Medicare HHA claims to OASIS based on beneficiary identifiers and the start-of-care date. Demographic characteristics from the MBSF, including race, and information about a patient’s diagnosis of Alzheimer disease and related dementias (ADRD) from the CCW were linked by beneficiary identifier and year. We used MedPAR to identify inpatient hospitalizations and Medicare outpatient claims to determine outpatient observational stays. We defined post–acute care patients receiving HHA care as individuals whose start-of-care date was within 14 days of the inpatient discharge date listed in MedPAR. We obtained star rating information and HHA characteristics from the CMS HHA Care Compare website from 2015 to 2019.^18^

### Study Design and Sample

We conducted patient spell–level analyses to compare annual changes in our measures over time. A patient spell was either an episode of HHA care from admission date to discharge date, or a combination of several episodes that were within 10 days of each other. The year of the spell was attributed based on the start-of-care date identified in the HHA claims, with 2020 data used to follow spells that began in 2019 but may have ended in 2020 (study flowchart in eFigure 1 in Supplement 1).

### Outcome Variables

The primary outcomes for this study included both process and outcome measures derived from the claims and self-reported by HHAs in OASIS. Following prior work by the Medicare Payment Advisory Commission (MedPAC), the hospitalization outcomes captured inpatient hospital admissions, readmissions, and outpatient observational stays that occurred during the spell or 30 days after HHA discharge.^1^ Changes in functional status were captured by OASIS measures for ambulation, bed transferring, and bathing, with improvement recorded if the value reported on the discharge assessment indicated less impairment relative to the start-of-care assessment.^18^ For a subsample of PAC patients, we also measured timely initiation of care, defined as an HHA spell with a start-of-care date listed in OASIS within 2 days of the inpatient discharge date recorded in MedPAR.^18^

### Statistical Analysis

#### Primary Analysis

First, we looked at annual trends in our outcome variables from 2013 to 2019. Then we used interrupted time series (ITS) analyses to compare changes in outcomes before (January 2013 to December 2015) and after (January 2016 to December 2019) the introduction of the HHA star ratings. Because there was a 6-month period (July to December 2015) when only 1 star rating (eg, quality of patient care) was introduced, we set the beginning of the postexposure period to January 2016 to assess the cumulative association of when both the quality of patient care and patient satisfaction star ratings were available. We used a linear time-trend variable (eg, year) to model the baseline pretrend, an indicator for the post–star ratings period to measure the mean level change, and an interaction between the 2 to measure changes in slope after the introduction of star ratings. In a sensitivity analysis, we used a specification which set the beginning of the postexposure period to July 2015, when only 1 star rating measure was available. We adjusted for autocorrelation using Newey-West standard erorrs.^19^ Because of the potential for type I errors due to multiple comparisons, we considered the interpretation of our results as exploratory. Statistical significance was assessed at *P* < .05 using 2-tailed tests. All analyses were conducted using SAS version 9.4 (SAS Institute) from November 2022 to September 2023.

#### Secondary Analysis

We also assessed changes over time for high-quality HHAs because we hypothesized that they might be associated with positive changes in both process measures (eg, greater timely initiation of care) and patient outcomes (eg, lower hospitalizations and greater functional improvement). Patient spells were attributed to a high-quality HHA if the HHA received 4 or 5 stars in the same reporting period as the spell start-of-care date for (1) quality of patient care, (2) patient satisfaction, or (3) both star ratings. The quality of patient care rating began in July 2015 and patient satisfaction in January 2016, with HHAs scored from 1 star (worse) to 5 stars (better). The quality of patient care rating is a composite measure capturing improvement in function, potentially avoidable events, utilization of care, and cost and/or resources, whereas the patient satisfaction rating is based on the HHA Consumer Assessment of Health Providers and Systems survey.^18^ Finally, we examined whether there were differential changes by patient characteristics, including race (Black, Hispanic, White, or other [Asian, American Indian, Alaska native, native Hawaiian, or Pacific Islander]), dual-eligibility status (eligible for both Medicare and Medicaid), and diagnosis of ADRD.

## Results

### Characteristics Before and After Star Ratings

There were a total of 22 958 847 patient spells included in the study from January 1, 2013, to December 31, 2019. Among 9 750 689 patient spells included during the pre–star ratings period from January 1, 2013, to December 31, 2015, 6 067 113 (62.2%) were female, 1 100 145 (11.3%) were Black, 512 487 (5.3%) were Hispanic, and 7 845 197 (80.5%) were White; 2 656 124 (27.2%) were dual eligible; 3 513 108 (36.0%) were diagnosed with ADRD; mean (SD) age was 77.4 (12.0) years. Among 13 208 158 patient spells included during the post–star ratings period from January 1, 2016, to December 31, 2019, 8 104 69 (61.4%) were female, 1 385 180 (10.5%) were Black, 675 536 (5.1%) were Hispanic, and 10 664 239 (80.7%) were White; 3 318 113 (25.1%) were dual eligible; 5 245 624 (39.7%) were diagnosed with ADRD; mean (SD) age was 77.7 (11.6) years. (Table 1). Patient and HHA characteristics before and after the introduction of star ratings by high-quality facilities are in eTable 1 in Supplement 1.

**Table 1.** Patient and HHA Characteristics Before and After the Introduction of Star Ratings^a^

Patient characteristics	Pre–star ratings, Jan 2013-Dec 2015		Post–star ratings, Jan 2016-Dec 2019	
	No.	% (SD)	No.	% (SD)
No. of patient spells	9 750 689	NA	13 208 158	NA
Age, mean (SD), y	NA	77.4 (12.0)	NA	77.7 (11.6)
Sex				
Male	3 683 576	37.8 (48.5)	5 103 465	38.6 (48.7)
Female	6 067 113	62.2 (48.5)	8 104 69	61.4 (48.7)
Race				
Black	1 100 145	11.3 (31.6)	1 385 180	10.5 (30.6)
Hispanic	512 487	5.3 (22.3)	675 536	5.1 (22.0)
White	7 845 197	80.5 (39.7)	10 664 239	80.7 (39.4)
Other race^b^	292 860	3.0 (17.1)	483 203	3.7 (18.8)
Dual eligible	2 656 124	27.2 (44.5)	3 318 113	25.1 (43.4)
ADRD	3 513 108	36.0 (48.0)	5 245 624	39.7 (48.9)
No. of comorbidities^c^	NA	7.41 (2.96)	NA	7.59 (2.97)
Postacute^d^	3 321 440	34.1 (47.4)	4 782 387	36.2 (48.1)
Patient spell duration, mean (SD), d	NA	70.9 (124.9)	NA	65.3 (96.2)
Home health agency characteristics^e^				
Nonprofit	3 754 345	38.5 (48.7)	4 705 221	35.6 (47.9)
For-profit	5 714 939	58.6 (49.3)	8 201 744	62.1 (48.5)
Government	281 405	2.9 (16.7)	301 193	2.3 (14.9)
No. of HHAs per patient zip code	NA	19.7 (25.2)	NA	24.4 (31.6)

Abbreviations: ADRD, Alzheimer disease and related dementias; HHA, home health agency; NA, not applicable.

^a^ Number of patient spells reported for characteristics listing percentages.

^b^ Other race included Asian, American Indian, Alaska Native, Native Hawaiian, or Pacific Islander.

^c^ Number of chronic comorbidities is based on the Chronic Conditions Warehouse (maximum = 27 comorbidities).

^d^ Patients receiving post–acute care HHA care was determined based on a home health admission within 14 days of an inpatient discharge.

^e^ The percentages reported for HHA characteristics are based on the number of patient spells.

### Overall Means of Outcome Measures

We first compared means over time for each measure (Table 2). The share of patient spells with a hospitalization increased slightly from 12.8% (409 733 of 3 214 297) in 2013 to 13.8% (451 317 of 3 267 868) in 2019. Similarly, the hospitalization rate within 30 days of discharge increased from 7.6% (244 785 of 3 214 297) in 2013 to 8.1% (265 483 of 3 267 868) in 2019, or a 0.5–percentage point increase. In this same period, the share of spells with timely initiation of care also declined, from 68.6% (764 191 of 1 114 085) in 2013 to 67.4% (773 169 of 1 146 935) in 2016 after the implementation of star ratings to 63.9% (821 961 of 1 285 601) by 2019, or a 4.7–percentage point decrease. In contrast, there were large changes in functional improvement reported by HHAs. The share of HHA patients with less impairment reported on an OASIS discharge assessment relative to the start-of-care assessment was an increase of 18.2 percentage points for ambulation, 24.4 percentage points for bed transferring, and 14.7 percentage points for bathing.

**Table 2.** Mean Outcomes Over Time Among Patients Receiving Care From Home Health Agencies, 2013-2019^a^

Outcome	2013	2014	2015	2016	2017	2018	2019	Percentage point change (2013 to 2019)
Hospitalization rate during spell^b^								
No. in sample	3 214 297	3 234 087	3 302 305	3 338 972	3 314 689	3 286 629	3 267 868	NA
No. in numerator (%)	409 733 (12.8)	413 000 (12.8)	415 354 (12.6)	421 165 (12.6)	428 037 (12.9)	434 652 (13.2)	451 317 (13.8)	1.0
Hospitalization rate within 30 d of discharge^b^								
No. in sample	3 214 297	3 234 087	3 302 305	3 338 972	3 314 689	3 286 629	3 267 868	NA
No. in numerator (%)	244 785 (7.6)	256 608 (7.9)	259 596 (7.9)	264 633 (7.9)	263 782 (8.0)	259 553 (7.9)	265 483 (8.1)	0.5
Timely initiation of care^c^								
No. in sample	1 114 085	1 095 477	1 111 878	1 146 935	1 164 599	1 185 252	1 285 601	NA
No. in numerator (%)	764 191 (68.6)	744 168 (67.9)	747 049 (67.2)	773 169 (67.4)	778 035 (66.8)	772 470 (65.2)	821 961 (63.9)	−4.7
Improvement in ambulation								
No. in sample	2 395 922	2 410 901	2 502 478	2 577 144	2 583 851	2 521 559	2 071 172	NA
No. in numerator (%)	1 526 263 (63.7)	1 588 897 (65.9)	1 743 348(69.7)	1 924 127 (74.7)	2 014 470 (78.0)	2 027 246 (80.4)	1 694 943 (81.8)	18.1
Improvement in bed transferring								
No. in sample	2 248 028	2 294 215	2 409 571	2 525 994	2 548 475	2 496 195	2 051 896	NA
No. in numerator (%)	1 317 549 (58.6)	1 393 981(60.76)	1 583 024(65.7)	1 807 052 (71.8)	1 975 028 (77.5)	2 022 832 (81.0)	1 719 920 (83.8)	25.2
Improvement in bathing								
No. in sample	2 443 518	2 447 415	2 530 971	2 597 500	2 599 091	2 533 708	2 080 914	NA
No. in numerator (%)	1 682 777(68.9)	1 715 377(70.1)	1 844 330 (72.9)	1 994 558 (76.8)	2 066 527 (79.5)	2 073 200 (81.8)	1 744 133 (83.8)	14.9

Abbreviation: NA, not applicable.

^a^ Number in sample represents the number of patient spells in the denominator. Number in sample varies depending on the outcome due to differences in the number of patient spells with a valid OASIS start of care and discharge assessment recording functional status (for the improvement in ambulation, bed transferring, and bathing measures). Number in the numerator represents the number of patient spells with each outcome.

^b^ Hospitalization rate variables included inpatient hospitalizations, readmissions, and outpatient observational visits.

^c^ Timely initiation of care refers to home health spells that started within 2 days of an inpatient discharge date. The timely initiation of care outcome was only measured among post–acute care home health agency users (eg, patient spells that started within 14 days of hospital discharge).

We also examined these differences by patient characteristics (eg, race, dual-eligibility status, and ADRD) (eTable 2 in Supplement 1), among patients who received their care from high-quality HHAs (eFigures 2 and 3 in Supplement 1), and the interaction between patient characteristics and quality status (eTables 3, 4, and 5 in Supplement 1). Despite different baseline levels, trends remained similar across measures and racial groups, with slightly larger increases in hospitalizations both during the spell and after discharge from the HHA observed among Black and Hispanic patients compared with the other race groups (eTable 2 in Supplement 1). These results were also consistent when we examined the interaction between high-quality status and patient characteristics (eTable 3 in Supplement 1).

### Rates and Trends of Outcome Measures

From our ITS model, we did not identify significant differences in trends before star ratings were introduced, levels after the introduction, or in slopes comparing the pre– to post–star ratings periods for the hospitalization measures (Table 3). However, the introduction of star ratings was associated with accelerations in the mean (SE) hospitalization rate during a spell (0.39% [0.05%] per year increase; *P* < .001). Mean (SE) rates of timely initiation of care slightly declined (−0.70% [0.18%] per year; *P* = .03) while functional improvement measures increased by 2.98% (0.46%) per year for ambulation (*P* = .01), 3.54% (0.76%) per year for bed transferring (*P* = .01), and 2.00% (0.30%) per year for bathing (*P* = .01) prior to the introduction of HHA star ratings. The introduction of star ratings was associated with a significant level change, as rates subsequently increased by 1.65% (0.40%) for timely initiation of care (*P* = .03) and 3.32% (1.00%) for functional improvement in ambulation (*P* = .04). After the introduction of star ratings, it was associated with a decline in timely initiation of care (−1.21% [0.12%] per year; *P* < .001) and increases in the functional improvement measures (2.40 [0.29%] per year for ambulation, 3.95% [0.48%] per year for bed transferring, and 2.34% [0.19%] per year for bathing; *P* < .001). In the sensitivity analysis which set the start of the postexposure period to July 2015, we observed that the coefficient estimates for the postexposure trend were similar in direction, but much smaller in magnitude compared with the main specification with January 2016 as the start of the postexposure period (eTable 6 in Supplement 1).

**Table 3.** Rates and Trends in Claims-Based and Home Health Agency-Reported Outcome Measures, 2013-2019

Outcome	Parameter^a^	Interpretation	Estimate (SE)	*P* value
Hospitalization rate during spell^b^	*β_1_*	Preexposure trend	−0.09 (0.07)	.33
	*β_2_*	Postexposure level change	−0.45 (0.16)	.06
	*β_3_*	Postexposure trend change	0.48 (0.09)	.12
	*β_1_* *+* *β_3_*	Postexposure trend	0.39 (0.05)	<.001
Hospitalization rate within 30 d of discharge^b^	*β_1_*	Preexposure trend	0.12 (0.08)	.24
	*β_2_*	Postexposure level change	−0.08 (0.18)	.68
	*β_3_*	Postexposure trend change	−0.07 (0.10)	.54
	*β_1_* *+* *β_3_*	Postexposure trend	0.05 (0.05)	.31
Timely initiation of care^c^	*β_1_*	Preexposure trend	−0.70 (0.18)	.03
	*β_2_*	Postexposure level change	1.65 (0.40)	.03
	*β_3_*	Postexposure trend change	−0.50 (0.22)	.10
	*β_1_* *+* *β_3_*	Postexposure trend	−1.21 (0.12)	<.001
Improvement in ambulation	*β_1_*	Preexposure trend	2.98 (0.46)	.01
	*β_2_*	Postexposure level change	3.32 (1.00)	.04
	*β_3_*	Postexposure trend change	−0.59 (0.55)	.36
	*β_1_* *+* *β_3_*	Postexposure trend	2.40 (0.29)	<.001
Improvement in bed transferring	*β_1_*	Preexposure trend	3.54 (0.76)	.02
	*β_2_*	Postexposure level change	3.43 (1.64)	.13
	*β_3_*	Postexposure trend change	0.41 (0.90)	.68
	*β_1_* *+* *β_3_*	Postexposure trend	3.95 (0.48)	<.001
Improvement in bathing	*β_1_*	Pre-exposure trend	2.00 (0.30)	.01
	*β_2_*	Postexposure level change	2.02 (0.65)	.05
	*β_3_*	Postexposure trend change	0.34 (0.36)	.41
	*β_1_* *+* *β_3_*	Postexposure trend	2.34 (0.19)	<.001

^a^ Parameters were estimated from an interrupted time series regression model of *Y* = α + *β_1_T* + *β_2_X* + *β_3_XT* + ε, where *t* = linear time and *X* = post–star ratings period. The pre–star ratings period was from January 2013 to December 2015 and the post–star ratings period was from January 2016 to December 2019.

^b^ Hospitalization rate variables included inpatient hospitalizations, readmissions, and outpatient observational visits.

^c^ Timely initiation of care refers to home health spells that started within 2 days of an inpatient discharge date.

Our results were similar when comparing outcomes among patients who received care from a high-quality HHA (quality of patient care, patient satisfaction, or both star ratings) (Table 4), with slightly larger accelerations in the hospitalization rate and less improvement observed in the functional measures for patients receiving care from an HHA that was high quality based on the quality of patient care or on both ratings compared with HHAs that were high quality based on the patient satisfaction star rating only. The introduction of the star ratings was also associated with sustained increases in the hospitalization rate and functional improvement measures observed among patients with ADRD, dual-eligible, Black race, and Hispanic race (Table 5). These patterns were also consistent when comparing outcomes among those who received care from high-quality HHAs by patient characteristics (eTable 7 in Supplement 1).

**Table 4.** Rates and Trends in Outcome Measures Among High-Quality Home Health Agencies, 2013-2019

Outcome	Parameter^a^	Interpretation	Patient quality of care		Patient satisfaction		Both star ratings	
			Estimate (SE)	*P* value	Estimate (SE)	*P* value	Estimate (SE)	*P* value
Hospitalization rate during spell^b^	*β_1_*	Preexposure trend	−0.06 (0.08)	.51	−0.07 (0.07)	.42	−0.07 (0.09)	.49
	*β_2_*	Postexposure level change	−0.32 (0.17)	.16	−0.35 (0.15)	.10	−0.18 (0.18)	.39
	*β_3_*	Postexposure trend change	0.87 (0.09)	.00	0.56 (0.08)	.01	0.94 (0.10)	.00
	*β_1_* *+* *β_3_*	Postexposure trend	0.81 (0.05)	<.001	0.49 (0.04)	<.001	0.87 (0.05)	<.001
Hospitalization rate within 30 d of discharge^b^	*β_1_*	Preexposure trend	0.12 (0.08)	.22	0.13 (0.08)	.19	0.10 (0.08)	.30
	*β_2_*	Postexposure level change	−0.01 (0.16)	.94	−0.10 (0.17)	.61	−0.01 (0.17)	.95
	*β_3_*	Postexposure trend change	0.04 (0.09)	.71	−0.03 (0.09)	.78	0.03 (0.09)	.80
	*β_1_* *+* *β_3_*	Postexposure trend	0.15 (0.05)	.00	0.10 (0.05)	.03	0.12 (0.05)	.01
Timely initiation of care^c^	*β_1_*	Preexposure trend	−0.57 (0.19)	.06	−0.65 (0.22)	.06	−0.45 (0.27)	.19
	*β_2_*	Postexposure level change	1.03 (0.41)	.09	2.31 (0.48)	.02	1.23 (0.58)	.12
	*β_3_*	Postexposure trend change	−0.97 (0.23)	.02	−0.79 (0.26)	.06	−1.02 (0.32)	.05
	*β_1_* *+* *β_3_*	Postexposure trend	−1.54 (0.12)	<.001	−1.44 (0.14)	<.001	−1.47 (0.17)	<.001
Improvement in ambulation	*β_1_*	Preexposure trend	2.64 (0.51)	.01	3.05 (0.52)	.01	2.65 (0.51)	.01
	*β_2_*	Postexposure level change	2.65 (1.10)	.09	3.38 (1.12)	.06	2.81 (1.09)	.08
	*β_3_*	Postexposure trend change	−0.72 (0.60)	.32	−0.65 (0.62)	.37	−0.73 (0.60)	.31
	*β_1_* *+* *β_3_*	Postexposure trend	1.93 (0.32)	<.001	2.40 (0.33)	<.001	1.92 (0.32)	<.001
Improvement in bed transferring	*β_1_*	Preexposure trend	3.14 (0.84)	.03	3.67 (0.87)	.02	3.20 (0.81)	.03
	*β_2_*	Postexposure level change	3.54 (1.82)	.15	3.44 (1.87)	.16	3.54 (1.74)	.14
	*β_3_*	Postexposure trend change	0.17 (1.00)	.87	0.30 (1.02)	.79	0.13 (0.95)	.90
	*β_1_* *+* *β_3_*	Postexposure trend	3.32 (0.53)	<.001	3.96 (0.55)	<.001	3.32 (0.51)	<.001
Improvement in bathing	*β_1_*	Preexposure trend	1.60 (0.27)	.01	2.05 (0.34)	.01	1.68 (0.27)	.01
	*β_2_*	Postexposure level change	1.35 (0.58)	.10	2.11 (0.74)	.06	1.60 (0.59)	.07
	*β_3_*	Postexposure trend change	0.37 (0.32)	.33	0.31 (0.40)	.49	0.28 (0.32)	.45
	*β_1_* *+* *β_3_*	Postexposure trend	1.97 (0.17)	<.001	2.37 (0.22)	<.001	1.96 (0.17)	<.001

^a^ Parameters were estimated from an interrupted time series regression model of *Y* = α + *β_1_T* + *β_2_X* + *β_3_XT* + ε, where *t* = linear time and *X* = post–star ratings period. The pre–star ratings period was from January 2013 to December 2015 and the post–star ratings period was from January 2016 to December 2019.

^b^ Hospitalization rate variables included inpatient hospitalizations, readmissions, and outpatient observational visits.

^c^ Timely initiation of care refers to home health spells that started within 2 days of an inpatient discharge date.

**Table 5.** Rates and Trends in Outcome Measures by Characteristics of Patients Receiving Care From Home Health Agencies, 2013-2019

Outcome	Parameter^a^	Interpretation	Dual eligible^b^		ADRD		Black		Hispanic		White		Other race^c^	
			Estimate	*P* value	Estimate	*P* value	Estimate	*P* value	Estimate	*P* value	Estimate	*P* value	Estimate	*P* value
Hospitalization rate during spell^d^	*β_1_*	Preexposure trend	−0.11	.32	−0.10	.37	−0.01	.97	−0.09	.20	−0.03	.77	−0.02	.85
	*β_2_*	Postexposure level change	−0.54	.07	−0.41	.14	−0.61	.14	−0.43	.04	−0.55	.08	−0.38	.16
	*β_3_*	Postexposure trend change	0.62	.01	0.50	.02	0.65	.03	0.61	.003	0.53	.02	0.47	.02
	*β_1_* *+* *β_3_*	Postexposure trend	0.51	<.001	0.40	<.001	0.64	<.001	0.52	<.001	0.50	<.001	0.45	<.001
Hospitalization rate within 30 d of discharge^d^	*β_1_*	Preexposure trend	0.17	.19	0.18	.15	0.20	.22	0.12	.33	0.15	.22	0.21	.06
	*β_2_*	Postexposure level change	−0.14	.58	0.02	.91	−0.26	.42	0.02	.93	−0.13	.58	−0.32	.12
	*β_3_*	Postexposure trend change	0.02	.90	−0.17	.23	0.11	.52	0.17	.25	−0.04	.72	−0.01	.95
	*β_1_* *+* *β_3_*	Postexposure trend	0.19	.004	0.01	.84	0.31	<.001	0.28	<.001	0.10	.09	0.21	<.001
Timely initiation of care^e^	*β_1_*	Preexposure trend	−1.12	.002	−1.14	.002	−1.10	.04	−1.48	.004	−0.71	.04	−0.55	.28
	*β_2_*	Postexposure level change	1.17	.01	0.52	.10	0.87	.28	1.23	.06	1.97	.02	1.73	.15
	*β_3_*	Postexposure trend change	−0.34	.07	−0.10	.47	−0.38	.37	0.25	.33	−0.70	.06	−0.94	.16
	*β_1_* *+* *β_3_*	Postexposure trend	−1.45	<.001	−1.24	<.001	−1.48	<.001	−1.22	<.001	−1.41	<.001	−1.49	<.001
Improvement in ambulation	*β_1_*	Preexposure trend	3.16	.01	3.11	.01	3.35	.004	3.03	.001	2.94	.01	2.40	.03
	*β_2_*	Postexposure level change	3.33	.05	3.86	.05	3.12	.04	2.86	.01	3.38	.05	3.08	.09
	*β_3_*	Postexposure trend change	−0.72	.31	−0.34	.63	−0.87	.18	−0.50	.19	−0.56	.39	−0.17	.82
	*β_1_* *+* *β_3_*	Postexposure trend	2.44	<.001	2.77	<.001	2.48	<.001	2.53	<.001	2.38	<.001	2.23	<.001
Improvement in bed transferring	*β_1_*	Preexposure trend	3.72	.02	3.87	.02	3.74	.02	3.82	.01	3.50	.02	3.31	.02
	*β_2_*	Postexposure level change	3.22	.15	4.03	.11	3.27	.14	2.55	.13	3.53	.12	2.55	.20
	*β_3_*	Postexposure trend change	0.37	.71	0.59	.59	0.47	.64	0.34	.64	0.41	.68	0.63	.51
	*β_1_* *+* *β_3_*	Postexposure trend	4.09	<.001	4.46	<.001	4.21	<.001	4.16	<.001	3.9	<.001	3.94	<.001
Improvement in bathing	*β_1_*	Preexposure trend	2.30	.01	2.47	.01	2.38	.01	2.24	<.001	1.93	.01	1.89	.01
	*β_2_*	Postexposure level change	2.11	.07	2.79	.06	1.86	.08	1.48	.01	2.09	.05	1.66	.07
	*β_3_*	Postexposure trend change	0.10	.83	0.73	.26	0.07	.87	0.14	.39	0.39	.37	0.44	.27
	*β_1_* *+* *β_3_*	Postexposure trend	2.40	<.001	3.19	<.001	2.45	<.001	2.38	<.001	2.32	<.001	2.33	<.001

Abbreviation: ADRD, Alzheimer disease and related dementias.

^a^ Parameters were estimated from an interrupted time series regression model of *Y* = α + *β_1_T* + *β_2_X* + *β_3_XT* + ε, where *t* = linear time and *X* = post–star ratings period. The pre–star ratings period was from January 2013 to December 2015 and the post–star ratings period was from January 2016 to December 2019.

^b^ Dual eligible indicates a patient who is dually eligible for Medicare and Medicaid.

^c^ Other race included Asian, American Indian, Alaska Native, Native Hawaiian, or Pacific Islander.

^d^ Hospitalization rate variables included both inpatient hospitalizations, readmissions, and outpatient observational visits.

^e^ Timely initiation of care refers to home health spells that started within 2 days of an inpatient discharge date.

## Discussion

Our study contributes to the existing literature by leveraging a longer time horizon to understand changes in HHA outcomes 4 years after the implementation of HHA star ratings and used both claims-based and agency-reported measures. We observed some undesirable trends over time, with higher hospitalization rates and less timely initiation of care. Yet, we also observed large improvement in patients’ functional status based on HHA-reported measures in OASIS during this same period. Though we mainly focused on overall trends, there were some differences in the observed levels of the outcomes, with Black, dual-eligible, and ADRD patients associated with higher levels of hospitalizations, lower timely initiation, and less functional improvement, regardless of receiving care from a high-quality agency. This aligned with prior work establishing that socially higher-risk patients are less likely to report high experience of care ratings^20^ and are more likely to experience adverse clinical outcomes.^21^

The similarity in trends between high quality of patient care and high quality patient satisfaction HHAs may be surprising given prior literature, which identified a weak correlation between these 2 ratings.^22,23^ One explanation might be because functional outcomes are strongly associated with patient experience, as patients may associate positive functional improvements in their ability to perform activities of daily living with the care they receive from an HHA, and thus, highly rate their care on patient satisfaction surveys.^22^

The observed functional improvement was dampened by corresponding increases in more objective measures, such as hospitalizations and declines in timely initiation of care. This raises concern about how HHA-reported outcomes should be interpreted and used to assess quality. Our findings are aligned with prior work by MedPAC, which found that between 2014 and 2018, HHAs reported improvements in function but not hospitalization rates or emergency department visits.^24^ As a result, MedPAC does not consider measures of patient functional improvement when assessing HHA quality due to concerns about its reliability.^24^ While functional status, but not functional improvement between HHA admission and discharge, is used to assign patients into case-mix groups for Medicare payments, HHAs still may have an indirect incentive to report positive improvement. Furthermore, prior literature has established a correlation between functional status and readmission rates, as functional improvement has consistently been associated with lower readmission rates across a variety of populations, including the US broadly,^25^ PAC settings,^26^ and HHAs specifically.^27^ Taken together, the association with increased hospitalization rates raises questions about whether HHAs have achieved meaningful quality improvement or have just reported to have done so, based on these agency-reported measures.

We also observed a decline in the timely initiation of care over time. Achieving timely initiation is crucial, with prior work identifying that delayed care was associated with higher rehospitalization rates.^28,29^ In particular, researchers have also noted an association by race and ADRD status, as Black and Hispanic patients with ADRD were more likely to experience delays in timely initiation of care.^30,31^ However, we acknowledge that it is difficult to attribute who is ultimately responsible for delayed care, as it could result from a lack of coordination and delays in referrals by the hospital or capacity and staffing constraints experienced by HHAs. It will be important for future work to unpack the drivers of these mechanisms to better understand how quality among HHAs is changing.

### Limitations

This study has limitations. First, we are not able to separate the associations identified from the introduction of star ratings for HHAs from other HHA quality initiatives, such as the 2016 pilot of the Home Health Value-Based Purchasing Model in 9 US states, which provided financial incentives for participating HHAs to improve quality of patient care.^32^ However, given the introduction of these 2 programs in the same years, we view the observed functional improvement in OASIS-based measures relative to claims-based measures even more salient. Second, our data do not allow us to capture more recent changes in Medicare HHA reimbursements, such as the 2020 Patient-Driven Grouping Model or 2022 nationwide expansion of the Home Health Value-Based Purchasing Model, which might contribute to changes in HHA behavior and performance.^1^ Third, our measure of timely initiation of care is based on the inpatient discharge date, which may differ from the physician start-of-care date or referral-to-HHA date. Additionally, a delayed initiation of care may be appropriate for some patients, such as those awaiting a change in weight-bearing status before initiating HHA care. Fourth, we attributed the timing of some outcomes based on CMS dates for public reporting. Although there is a delay between the collection of data and public reporting, we believe that HHAs would be more responsive to these measures once public reporting began. Additionally, we attributed patient spells to high-quality HHAs based on their admission date, but quality may have not been consistent across the entire period for longer patient spells. We also excluded HHAs that were missing star ratings. Because there is a minimum number of episodes and/or responses needed to receive a star rating, the excluded agencies tend to be smaller HHAs. Additionally, some outcomes were part of the composite measure used to calculate an HHA’s quality of patient care star rating. Although an HHA’s performance on 1 of these measures is not deterministic of their rating, it may have been correlated. However, our results were consistent when we examined the outcomes by the patient satisfaction measure, which does not directly incorporate the outcomes in its rating calculation.

## Conclusion

This cross-sectional study assessing changes in HHA patient outcomes found that the introduction of the star ratings program was associated with higher hospitalization rates, decreased timely initiation of care, and increased functional improvement. These trends were consistent regardless of receiving care from a high-quality HHA and did not vary by patient characteristics. The observed improvement in agency-reported functional measures alongside declines in more objective, claims-based measures raise concern about using agency-reported measures to assess HHA quality. Thus, evaluating HHA quality of care will require a critical lens to understand how to interpret changes in objective compared with subjective measures.

## Notes

Supplement 1. **eTable 1. **Patient and HHA Characteristics Before and After the Introduction of Home Health Compare Star Ratings by High Quality Facilities **eTable 2. **Trends in Outcomes by Race, Dual-Eligible Status, and ADRD (2013-2019) **eTable 3. **Trends in Patient Outcomes Among High Quality HHAs by Race (2013-2019) **eTable 4. **Trends in Patient Outcomes Among High Quality HHAs by Dual-Eligible Status (2013-2019) **eTable 5. **Trends in Patient Outcomes Among High Quality HHAs by ADRD Status (2013-2019) **eTable 6. **Rates and Trends in Claims-Based and Home Health Agency-Reported Outcome Measures: Interrupted Time Series Analysis With a Post-Period Start Date of July 2015 (2013-2019) **eTable 7. **Rates and Trends in Patient Outcomes Among High Quality HHAs (Both Star Ratings) by Patient Characteristics (2013-2019) **eFigure 1. **Study Flowchart **eFigure 2. **Trends in Claims-Based Outcomes by High Quality HHA Status **eFigure 3. **Trends in OASIS-Based Outcomes by High Quality HHA Status

Supplement 2.
Data Sharing Statement
