# Supplementary material for: Claims-Based vs Agency-Reported Patient Outcomes Among Home Health Agencies, 2013-2019
Source: JAMA Netw Open. 2024 Apr 10;7(4):e245692. doi: 10.1001/jamanetworkopen.2024.5692 (PMC11007578; doi:10.1001/jamanetworkopen.2024.5692)
Supplement: Supplement 1. — eTable 1. Patient and HHA Characteristics Before and After the Introduction of Home Health Compare Star Ratings by High Quality Facilities eTable 2. Trends in Outcomes by Race, Dual-Eligible Status, and ADRD (2013-2019) eTable 3. Trends in Patient Outcomes Among High Quality HHAs by Race (2013-2019) eTable 4. Trends in Patient Outcomes Among High Quality HHAs by Dual-Eligible Status (2013-2019) eTable 5. Trends in Patient Outcomes Among High Quality HHAs by ADRD Status (2013-2019) eTable 6. Rates and Trends in Claims-Based and Home Health Agency-Reported Outcome Measures: Interrupted Time Series Analysis With a Post-Period Start Date of July 2015 (2013-2019) eTable 7. Rates and Trends in Patient Outcomes Among High Quality HHAs (Both Star Ratings) by Patient Characteristics (2013-2019) eFigure 1. Study Flowchart eFigure 2. Trends in Claims-Based Outcomes by High Quality HHA Status eFigure 3. Trends in OASIS-Based Outcomes by High Quality HHA Status [file jamanetwopen-e245692-s001.pdf]

Supplemental Online Content

Chen AC, Fu CX, Grabowski DC. Claims-based vs agency-reported patient outcomes among home health agencies, 2013-2019. *JAMA Netw Open*. 2024;7(4):e245692. doi:10.1001/jamanetworkopen.2024.5692

eTable 1. Patient and HHA Characteristics Before and After the Introduction of Home Health Compare Star Ratings by High Quality Facilities
eTable 2. Trends in Outcomes by Race, Dual-Eligible Status, and ADRD (2013-2019)
eTable 3. Trends in Patient Outcomes Among High Quality HHAs by Race (2013-2019)
eTable 4. Trends in Patient Outcomes Among High Quality HHAs by Dual-Eligible Status (2013-2019)
eTable 5. Trends in Patient Outcomes Among High Quality HHAs by ADRD Status (2013-2019)
eTable 6. Rates and Trends in Claims-Based and Home Health Agency-Reported Outcome Measures: Interrupted Time Series Analysis With a Post-Period Start Date of July 2015 (2013-2019)
eTable 7. Rates and Trends in Patient Outcomes Among High Quality HHAs (Both Star Ratings) by Patient Characteristics (2013-2019)
eFigure 1. Study Flowchart
eFigure 2. Trends in Claims-Based Outcomes by High Quality HHA Status
eFigure 3. Trends in OASIS-Based Outcomes by High Quality HHA Status

This supplemental material has been provided by the authors to give readers additional information about their work.

© 2024 Chen AC et al. *JAMA Network Open*.

eTable 1. Patient and HHA characteristics before and after the introduction of Home Health Compare Star Ratings by high quality facilities

	Year	Age	Male	Black	Hispanic	White	Other Race	Dual Eligible	ADRD	Post-Acute	Non-profit	For-profit	Government	Spell Length
**High Quality** (patient care)	2013	77.35	37.90	11.50	7.90	76.90	3.60	30.70	35.90	32.90	36.60	60.50	2.80	64.37
	2014	77.33	38.30	11.20	9.00	76.00	3.90	31.50	36.60	31.70	35.70	61.60	2.70	63.68
	2015	77.59	38.40	10.80	8.00	77.10	4.10	30.20	37.70	31.80	35.60	61.90	2.50	63.36
	2016	77.61	38.50	11.10	6.70	78.30	3.90	28.60	39.30	33.10	33.60	64.40	2.00	63.02
	2017	77.75	38.80	10.70	5.70	79.70	3.80	27.80	40.90	33.30	26.90	71.90	1.20	66.84
	2018	77.93	38.70	10.60	6.00	79.50	3.90	28.00	42.20	33.40	22.80	75.90	1.30	67.41
	2019	78.13	38.60	11.10	6.00	78.80	4.00	28.60	43.70	35.80	21.40	77.10	1.40	65.71
**High quality** (patient satisfaction)	2013	77.13	37.60	10.70	4.50	82.70	2.10	27.20	34.20	36.40	41.10	55.20	3.80	73.24
	2014	77.12	38.00	10.50	4.60	82.60	2.20	27.10	34.40	35.40	40.60	55.70	3.70	72.58
	2015	77.28	38.20	10.40	4.50	82.70	2.40	26.30	35.50	35.00	40.30	56.10	3.60	71.28
	2016	77.22	38.40	10.50	4.00	83.20	2.30	26.00	37.40	35.60	40.50	56.20	3.40	69.48
	2017	77.30	38.70	10.40	3.70	83.40	2.40	25.90	38.10	36.50	40.30	56.60	3.10	69.92
	2018	77.46	38.80	10.20	3.80	83.50	2.60	25.20	38.60	37.30	38.90	58.30	2.70	68.27
	2019	77.81	38.70	10.10	4.10	83.00	2.80	25.10	40.30	39.70	36.90	60.40	2.70	64.10
**High Quality** (both measures)	2013	77.42	38.00	9.30	4.90	83.60	2.20	26.20	33.80	36.30	43.00	53.40	3.60	63.38
	2014	77.44	38.40	9.20	5.20	83.30	2.30	26.30	34.30	35.40	42.30	54.20	3.50	63.04
	2015	77.62	38.50	9.00	4.90	83.70	2.40	25.40	35.30	35.10	41.90	54.80	3.30	62.59
	2016	77.42	38.60	9.90	4.00	83.70	2.40	25.10	36.70	36.00	40.40	56.80	2.80	63.61
	2017	77.45	38.80	10.20	3.40	84.10	2.30	25.30	38.30	35.90	32.70	65.60	1.80	68.67
	2018	77.60	38.90	10.10	3.60	83.90	2.40	24.80	39.30	36.10	27.70	70.80	1.50	68.96
	2019	77.90	38.60	10.60	4.00	82.90	2.60	25.30	41.40	38.10	24.70	73.60	1.70	67.48

Notes: Mean is reported for age. All other characteristics are reported as percentages (%). ADRD is Alzheimer's disease and related dementias. Other race included Asian, American Indian, Alaska native, native Hawaiian, or Pacific Islander.

© 2024 Chen AC et al. *JAMA Network Open*.

eTable 2. Trends in outcomes by race, dual-eligible status, and ADRD (2013-2019)

	Year	Hospitalization During Spell	Hospitalization Post-Discharge	Timely Initiation of Care	Ambulation	Bed transferring	Bathing
**By Race**							
**Black**	**2013**	13.88	7.78	64.46	58.38	53.07	65.40
	**2014**	13.90	8.05	63.64	60.95	54.84	66.67
	**2015**	13.84	8.15	62.41	64.65	59.42	69.47
	**2016**	14.02	8.41	61.51	69.59	65.17	73.22
	**2017**	14.45	8.67	60.91	72.95	70.89	76.03
	**2018**	15.02	8.79	58.64	75.60	74.85	78.56
	**2019**	15.88	9.47	57.47	77.56	78.44	80.85
**Hispanic**	**2013**	10.80	5.95	66.81	58.33	51.64	68.49
	**2014**	11.32	6.31	65.53	61.11	53.08	68.80
	**2015**	11.74	6.60	64.23	63.48	56.84	69.51
	**2016**	12.01	7.04	63.86	67.56	61.06	72.50
	**2017**	12.82	7.33	63.23	69.96	65.72	73.94
	**2018**	13.15	7.52	61.57	72.88	68.95	76.38
	**2019**	13.76	7.97	60.44	73.93	72.70	79.16
**White**	**2013**	12.99	7.55	68.66	63.34	58.57	68.73
	**2014**	12.96	7.90	67.94	65.47	60.51	69.84
	**2015**	12.84	7.84	67.20	69.30	65.32	72.62
	**2016**	12.90	7.91	67.46	74.27	71.30	76.52
	**2017**	13.20	7.95	66.71	77.58	76.81	79.22
	**2018**	13.59	7.95	64.96	80.00	80.17	81.50
	**2019**	14.34	8.26	63.48	81.48	83.16	83.65
**Other**	**2013**	10.68	6.15	66.91	61.54	54.80	67.11
	**2014**	10.55	6.32	66.95	63.13	56.02	68.10
	**2015**	10.40	6.37	65.79	65.92	59.60	70.43
	**2016**	10.51	6.40	65.77	70.13	64.48	73.92
	**2017**	10.89	6.53	65.33	73.75	69.29	76.33
	**2018**	11.11	6.57	63.43	75.64	71.91	78.13
	**2019**	11.69	6.89	61.63	77.00	75.37	81.10
**By Dual-Eligible Status**							
**Dual**	**2013**	14.00	7.86	65.99	57.73	51.98	64.97
	**2014**	14.07	8.19	64.94	60.22	53.55	65.90
	**2015**	14.02	8.26	63.81	63.72	58.02	68.44
	**2016**	14.10	8.48	63.44	68.48	63.20	72.14
	**2017**	14.50	8.57	62.33	71.65	68.39	74.67
	**2018**	14.81	8.63	60.53	74.12	71.30	76.91
	**2019**	15.49	9.09	59.22	75.36	74.84	79.29
**By Alzheimer's Disease and Related Dementias**							
**ADRD**	**2013**	15.11	9.34	65.53	54.21	49.42	57.01

	**2014**	15.05	9.72	64.59	56.31	51.31	58.01
	**2015**	14.94	9.71	63.29	60.36	56.50	61.45
	**2016**	15.04	9.86	62.71	65.94	63.13	66.57
	**2017**	15.33	9.84	61.46	69.54	69.05	69.83
	**2018**	15.57	9.78	60.00	72.33	72.88	72.98
	**2019**	16.23	9.99	59.16	74.08	76.46	76.08

Notes: ADRD is Alzheimer's disease and related dementias. Other race included Asian, American Indian, Alaska native, native Hawaiian, or Pacific Islander. All outcomes are reported as rates (%). Hospitalization rate variables included both in-patient hospitalizations, readmissions, and outpatient observational visits. Hospitalization rate post-discharge is measured within 30 days of HHA discharge. Timely initiation of care refers to home health spells which started within two days of an inpatient discharge. Ambulation, bed transferring, and ambulation are measures of functional improvement reported in OASIS.

eTable 3. Trends in patient outcomes among high quality HHAs by race (2013-2019)

	White							Black						
**year**	**2013**	**2014**	**2015**	**2016**	**2017**	**2018**	**2019**	**2013**	**2014**	**2015**	**2016**	**2017**	**2018**	**2019**
**High Quality (patient care)**														
Hospitalization during spell (%)	12.03	11.85	11.82	12.18	13.06	13.60	14.58	12.61	12.46	12.51	13.33	14.42	15.25	16.54
Hospitalization post-discharge (%)	7.27	7.50	7.50	7.64	7.78	7.79	8.09	7.31	7.64	7.66	8.09	8.44	8.58	9.27
Timely initiation (%)	70.70	70.51	69.73	69.28	67.97	66.34	64.70	65.96	66.40	64.13	62.86	62.45	60.29	59.07
Ambulation (%)	70.78	73.95	75.94	80.11	83.25	85.01	86.02	68.42	71.68	73.54	77.62	81.41	83.13	84.07
Bed transferring (%)	65.40	68.41	71.31	77.03	82.52	85.12	87.36	63.59	65.81	68.30	73.65	80.06	82.84	84.70
Bathing (%)	75.70	77.64	78.70	81.96	84.53	86.30	88.01	75.43	77.55	78.30	80.93	83.82	85.32	86.77
**High Quality (patient satisfaction)**														
Hospitalization during spell (%)	13.43	13.38	13.22	13.43	13.90	14.27	14.92	15.32	15.33	15.17	15.32	15.93	16.41	17.26
Hospitalization post-discharge (%)	7.58	7.92	7.86	7.94	8.01	8.03	8.22	8.16	8.50	8.58	8.65	8.77	8.93	9.53
Timely initiation (%)	69.18	68.52	67.92	68.54	67.76	66.06	64.30	65.93	65.42	64.41	63.83	63.16	61.12	59.86
Ambulation (%)	65.48	67.60	71.41	76.37	79.86	82.26	83.49	62.33	65.28	69.14	74.35	78.01	80.55	82.17
Bed transferring (%)	60.41	62.50	67.55	73.54	79.41	83.02	85.41	56.66	59.10	64.48	70.76	77.04	81.03	83.78
Bathing (%)	70.40	71.56	74.35	78.30	81.21	83.53	85.32	67.49	69.17	72.43	76.41	79.60	82.17	84.08
**High Quality (both)**														
Hospitalization during spell (%)	12.30	12.14	12.09	12.69	13.77	14.38	15.33	14.02	13.97	13.92	14.74	15.86	16.67	18.11
Hospitalization post-discharge (%)	7.30	7.57	7.52	7.64	7.81	7.79	8.01	7.82	8.27	8.19	8.39	8.50	8.46	9.16
Timely initiation (%)	71.17	71.08	70.33	70.01	69.03	67.43	65.52	67.26	67.95	65.92	64.61	64.11	62.59	61.13
Ambulation (%)	71.92	74.96	77.08	81.43	84.58	86.39	87.20	69.52	72.96	75.45	79.84	83.52	85.18	86.32
Bed transferring (%)	66.65	69.64	72.83	78.67	84.21	87.07	88.81	64.73	67.86	71.26	76.88	83.12	86.02	87.57
Bathing (%)	76.39	78.30	79.63	83.16	85.71	87.58	89.02	74.88	77.10	78.91	82.17	85.15	86.81	88.22

	Hispanic							Other Race						
**year**	**2013**	**2014**	**2015**	**2016**	**2017**	**2018**	**2019**	**2013**	**2014**	**2015**	**2016**	**2017**	**2018**	**2019**
**High Quality (patient care)**														
Hospitalization during spell (%)	9.43	8.39	8.75	9.79	11.35	11.74	12.80	9.51	9.24	9.06	9.20	10.18	10.58	11.32
Hospitalization post-discharge (%)	5.95	5.43	5.79	6.46	6.99	7.26	7.90	5.69	5.72	5.76	5.80	6.13	6.25	6.86
Timely initiation (%)	68.03	67.40	65.34	65.04	64.73	63.55	62.17	68.51	69.34	67.42	67.53	66.66	64.18	62.66
Ambulation (%)	68.60	73.80	74.83	77.50	79.22	81.64	81.14	69.85	72.05	72.15	76.12	80.93	82.67	83.74
Bed transferring (%)	59.67	63.38	67.07	70.64	74.91	76.93	80.20	62.06	63.48	64.99	69.89	76.77	78.12	82.20
Bathing (%)	77.45	82.02	80.94	81.76	82.74	84.72	86.54	75.10	77.20	77.37	80.19	83.12	84.60	87.46
**High Quality (patient satisfaction)**														
Hospitalization during spell (%)	13.93	13.74	13.77	13.81	14.57	14.55	14.90	12.27	12.23	12.06	12.34	12.87	12.80	13.19
Hospitalization post-discharge (%)	6.98	7.16	7.17	7.59	7.83	7.78	8.25	6.66	7.00	6.96	7.06	7.04	7.14	7.41
Timely initiation (%)	67.95	66.49	65.28	65.53	65.04	63.68	62.19	67.23	67.36	66.30	68.10	67.16	65.88	63.33
Ambulation (%)	60.46	63.46	66.82	71.88	74.77	78.09	79.98	65.73	68.24	71.70	76.34	79.28	81.22	81.75
Bed transferring (%)	53.72	56.37	62.01	68.26	73.77	77.36	80.66	59.69	62.41	66.95	72.18	77.19	80.56	82.75
Bathing (%)	66.25	68.82	70.82	74.62	77.58	80.17	82.81	69.89	71.57	74.11	77.84	80.57	83.00	84.31
**High Quality (both)**														
Hospitalization during spell (%)	10.91	10.41	10.53	11.73	12.92	13.23	14.11	10.73	10.66	10.59	10.89	12.06	12.33	13.05
Hospitalization post-discharge (%)	6.48	6.30	6.51	7.09	7.60	7.31	8.03	6.16	6.38	6.25	6.49	6.68	6.80	7.21
Timely initiation (%)	69.47	68.93	67.22	67.11	66.76	66.07	63.25	69.81	69.96	69.35	68.64	68.31	67.02	64.91
Ambulation (%)	70.71	74.68	75.99	80.23	81.71	84.31	85.38	72.21	75.77	77.87	81.20	84.33	85.82	86.95
Bed transferring (%)	62.93	66.18	71.26	77.38	82.29	83.99	85.73	65.01	68.30	71.40	76.08	82.20	84.31	86.75
Bathing (%)	77.14	80.99	80.41	82.02	84.38	86.41	88.13	76.38	79.16	80.78	82.67	85.94	87.65	89.40

Notes: HHA is home health agency. Other race included Asian, American Indian, Alaska native, native Hawaiian, or Pacific Islander.

eTable 4. Trends in patient outcomes among high quality HHAs by dual-eligible status (2013-2019)

	Non Dual-Eligible							Dual-Eligible						
**year**	**2013**	**2014**	**2015**	**2016**	**2017**	**2018**	**2019**	**2013**	**2014**	**2015**	**2016**	**2017**	**2018**	**2019**
**High Quality (patient care)**														
Hospitalization during spell (%)	12.37	12.19	12.18	12.49	13.36	13.97	14.97	10.01	9.46	9.49	10.41	11.67	12.07	13.10
Hospitalization post-discharge (%)	7.64	7.90	7.92	8.06	8.20	8.24	8.60	5.43	5.31	5.34	5.73	6.08	6.17	6.62
Timely initiation (%)	70.51	70.35	69.46	69.06	67.86	66.14	64.48	67.69	67.70	65.90	64.49	63.76	62.28	61.06
Ambulation (%)	71.01	74.06	76.02	80.16	83.35	85.08	86.07	68.13	72.32	73.62	77.29	80.55	82.54	83.12
Bed transferring (%)	65.93	68.99	71.93	77.55	83.13	85.81	87.76	60.53	62.99	65.50	70.48	76.16	78.13	81.67
Bathing (%)	75.89	77.79	78.76	81.97	84.57	86.34	87.98	75.48	78.66	78.85	81.06	83.32	84.94	87.04
**High Quality (patient satisfaction)**														
Hospitalization during spell (%)	13.68	13.64	13.51	13.70	14.19	14.50	15.19	13.47	13.32	13.06	13.28	13.78	14.30	14.72
Hospitalization post-discharge (%)	7.89	8.28	8.23	8.32	8.40	8.37	8.62	6.43	6.55	6.47	6.58	6.63	6.88	7.10
Timely initiation (%)	69.19	68.52	67.86	68.44	67.73	66.03	64.27	66.52	65.90	65.10	65.29	64.28	62.52	60.99
Ambulation (%)	65.91	68.03	71.77	76.62	80.04	82.52	83.68	61.10	63.84	67.80	73.35	77.15	79.30	80.93
Bed transferring (%)	60.84	62.94	67.94	73.87	79.68	83.36	85.62	55.28	57.63	63.03	69.47	75.70	79.07	82.21
Bathing (%)	70.77	71.93	74.62	78.49	81.35	83.73	85.46	66.53	68.27	71.44	75.71	79.04	81.23	83.35
**High Quality (both)**														
Hospitalization during spell (%)	12.59	12.43	12.38	12.94	14.00	14.60	15.61	11.46	11.20	11.15	12.27	13.51	14.14	15.13
Hospitalization post-discharge (%)	7.62	7.92	7.86	7.99	8.18	8.12	8.40	5.93	6.00	5.96	6.26	6.43	6.54	6.89
Timely initiation (%)	71.15	71.01	70.28	69.97	69.02	67.46	65.47	68.36	68.88	66.93	65.73	65.22	63.73	62.24
Ambulation (%)	72.28	75.27	77.42	81.63	84.66	86.51	87.26	69.13	72.85	74.75	79.51	83.15	84.78	86.08
Bed transferring (%)	67.27	70.34	73.61	79.21	84.59	87.48	88.98	62.24	65.03	68.32	74.88	81.49	83.81	86.44
Bathing (%)	76.65	78.53	79.86	83.30	85.75	87.66	89.02	74.89	77.66	78.71	81.81	85.04	86.62	88.48

Notes: HHA is home health agency.

eTable 5. Trends in patient outcomes among high quality HHAs by ADRD status (2013-2019)

	**Non Alzheimer's disease and related dementias**							**Alzheimer's disease and related dementias**						
**year**	**2013**	**2014**	**2015**	**2016**	**2017**	**2018**	**2019**	**2013**	**2014**	**2015**	**2016**	**2017**	**2018**	**2019**
**High Quality (patient care)**														
Hospitalization during spell (%)	10.65	10.41	10.36	10.72	11.59	12.18	13.21	13.86	13.42	13.48	14.05	15.03	15.43	16.30
Hospitalization post-discharge (%)	6.03	6.13	6.14	6.24	6.45	6.52	6.90	9.03	9.21	9.25	9.54	9.62	9.52	9.79
Timely initiation (%)	71.06	70.95	70.24	70.09	69.11	67.29	65.39	67.34	67.04	65.35	64.14	62.76	61.67	61.02
Ambulation (%)	74.44	77.63	79.51	83.50	86.59	88.31	89.13	62.50	66.19	68.24	72.83	76.65	78.88	80.19
Bed transferring (%)	68.99	71.67	74.47	79.71	85.08	87.41	89.42	56.49	59.82	63.24	69.73	76.13	79.22	82.23
Bathing (%)	81.19	83.28	83.98	86.66	88.93	90.28	91.57	65.48	68.19	69.49	73.54	76.97	79.69	82.39
**High Quality (patient satisfaction)**														
Hospitalization during spell (%)	12.25	12.22	12.06	12.08	12.49	12.95	13.61	16.30	16.16	15.91	16.19	16.74	16.87	17.31
Hospitalization post-discharge (%)	6.49	6.77	6.66	6.62	6.72	6.78	7.11	9.72	10.13	10.10	10.25	10.24	10.18	10.15
Timely initiation (%)	69.76	69.09	68.68	69.59	69.09	67.26	65.42	66.04	65.30	64.00	63.77	62.46	61.22	60.16
Ambulation (%)	69.28	71.56	75.30	80.18	83.49	85.78	87.05	56.01	58.22	62.47	68.28	72.29	75.14	76.82
Bed transferring (%)	64.00	66.09	70.98	76.78	82.36	85.73	88.05	51.08	53.37	59.18	66.18	72.78	77.02	79.99
Bathing (%)	75.87	77.13	79.67	83.27	85.82	87.75	89.30	57.48	58.89	62.70	68.14	72.06	75.37	78.12
**High Quality (both)**														
Hospitalization during spell (%)	11.14	10.99	10.87	11.39	12.34	12.97	14.02	14.75	14.48	14.47	15.26	16.44	16.90	17.63
Hospitalization post-discharge (%)	6.20	6.37	6.27	6.34	6.53	6.57	6.90	9.40	9.79	9.76	9.94	9.97	9.75	9.83
Timely initiation (%)	71.57	71.53	70.93	70.89	70.18	68.49	66.37	68.31	68.20	66.58	65.27	64.04	63.14	62.03
Ambulation (%)	75.74	78.77	80.89	85.06	87.98	89.57	90.48	63.12	66.56	68.98	73.98	77.97	80.43	81.65
Bed transferring (%)	70.47	73.37	76.49	81.88	87.16	89.62	91.19	57.65	60.95	64.92	71.83	78.39	81.99	84.31
Bathing (%)	81.63	83.59	84.69	87.66	89.89	91.28	92.41	65.03	67.43	69.50	74.21	78.00	80.98	83.45

Notes: HHA is home health agency. ADRD is Alzheimer's disease and related dementias.

eTable 6. Rates and trends in claims-based and home health agency-reported outcome measures: Interrupted time series analysis with a post-period start date of July 2015 (2013-2019)

outcome	Parameter	interpretation	estimate	standard error	p-value
**Hospitalization rate during spell**	$\beta_!$	pre-trend	-0.0003	0.001	0.61
	$\beta_"$	post-level change	-0.004	0.002	0.07
	$\beta_\#$	post-trend change	0.002	0.001	0.01
	$\beta_! + \beta_\#$	post-trend	0.002	0.0003	<.001
**Hospitalization rate within 30 days of discharge**	$\beta_!$	pre-trend	0.001	0.001	0.13
	$\beta_"$	post-level change	-0.001	0.002	0.48
	$\beta_\#$	post-trend change	-0.001	0.001	0.31
	$\beta_! + \beta_\#$	post-trend	0.0003	0.0002	0.20
**Timely initiation of care**	$\beta_!$	pre-trend	-0.004	0.001	0.02
	$\beta_"$	post-level change	0.01	0.005	0.03
	$\beta_\#$	post-trend change	-0.001	0.002	0.43
	$\beta_! + \beta_\#$	post-trend	-0.01	0.001	<.001
**Improvement in ambulation**	$\beta_!$	pre-trend	0.01	0.002	<.001
	$\beta_"$	post-level change	0.03	0.008	0.003
	$\beta_\#$	post-trend change	0.001	0.003	0.62
	$\beta_! + \beta_\#$	post-trend	0.01	0.001	<.001
**Improvement in bed transferring**	$\beta_!$	pre-trend	0.01	0.003	0.001
	$\beta_"$	post-level change	0.04	0.010	0.01
	$\beta_\#$	post-trend change	0.01	0.003	0.04
	$\beta_! + \beta_\#$	post-trend	0.02	0.001	<.001
**Improvement in bathing**	$\beta_!$	pre-trend	0.01	0.001	<.001
	$\beta_"$	post-level change	0.02	0.005	0.001
	$\beta_\#$	post-trend change	0.005	0.001	0.01
	$\beta_! + \beta_\#$	post-trend	0.01	0.001	<.001

Notes: hospitalization rate variables included both in-patient hospitalizations, readmissions, and outpatient observational visits; timely initiation of care refers to home health spells which started within two days of an inpatient discharge. Parameters are estimated from an ITS regression model of $Y = \alpha + \beta_! T + \beta_" X + \beta_\# XT + \varepsilon$ where T = linear time and X = post-star ratings period. The pre-star ratings period was from January 2013 to July 2015 and the post-star ratings period was from August 2015 to December 2019. The unit of analysis is half-years (6 months).

eTable 7. Rates and trends in patient outcomes among high quality HHAs (both star ratings) by patient characteristics (2013-2019)

			Dual		ADRD		Black		Hispanic		Other race		White	
**outcome**	**Parameter**	**interpretation**	**estimate**	**p-value**	**estimate**	**p-value**	**estimate**	**p-value**	**estimate**	**p-value**	**estimate**	**p-value**	**estimate**	**p-value**
**Hospitalization rate during spell**	$\beta_{!}$	pre-trend	-0.10	0.45	-0.10	0.45	-0.05	0.76	-0.17	0.41	-0.11	0.59	-0.06	0.52
	$\beta_{"}$	post-level change	0.15	0.57	0.16	0.57	-0.43	0.25	0.56	0.25	-0.17	0.70	-0.23	0.26
	$\beta_{\#}$	post-trend change	1.10	0.004	0.86	0.01	1.15	0.01	0.94	0.02	0.79	0.04	0.91	0.002
	$\beta_{!} + \beta_{\#}$	post-trend	1.00	<.001	0.75	<.001	1.11	<.001	0.77	<.001	0.68	<.001	0.85	<.001
**Hospitalization rate within 30 days of discharge**	$\beta_{!}$	pre-trend	0.14	0.25	0.17	0.17	0.14	0.49	0.01	0.95	0.04	0.65	0.10	0.24
	$\beta_{"}$	post-level change	0.10	0.67	0.21	0.38	-0.19	0.66	0.33	0.48	-0.16	0.43	-0.02	0.90
	$\beta_{\#}$	post-trend change	0.03	0.79	-0.24	0.12	0.07	0.76	0.26	0.32	0.19	0.15	0.00	0.99
	$\beta_{!} + \beta_{\#}$	post-trend	0.17	0.005	-0.07	0.26	0.22	0.06	0.27	0.02	0.23	<.001	0.10	0.02
**Timely initiation of care**	$\beta_{!}$	pre-trend	-0.66	0.22	-0.84	0.05	-0.61	0.33	-1.19	0.13	-0.11	0.84	-0.42	0.17
	$\beta_{"}$	post-level change	-0.31	0.76	-0.57	0.39	-0.27	0.83	1.63	0.28	0.83	0.49	1.38	0.07
	$\beta_{\#}$	post-trend change	-0.55	0.36	-0.23	0.52	-0.62	0.39	-0.07	0.92	-1.07	0.16	-1.08	0.03
	$\beta_{!} + \beta_{\#}$	post-trend	-1.22	<.001	-1.06	<.001	-1.23	<.001	-1.26	0.001	-1.18	<.001	-1.50	<.001
**Improvement in ambulation**	$\beta_{!}$	pre-trend	2.89	0.02	2.96	0.02	3.07	0.01	2.74	0.01	2.97	0.01	2.59	0.01
	$\beta_{"}$	post-level change	2.84	0.12	3.02	0.10	2.79	0.12	1.92	0.18	1.57	0.20	2.89	0.08
	$\beta_{\#}$	post-trend change	-0.78	0.36	-0.43	0.59	-0.96	0.27	-0.97	0.21	-1.09	0.13	-0.68	0.34
	$\beta_{!} + \beta_{\#}$	post-trend	2.11	<.001	2.53	<.001	2.11	<.001	1.78	<.001	1.88	<.001	1.91	<.001
**Improvement in bed transferring**	$\beta_{!}$	pre-trend	3.17	0.04	3.69	0.03	3.33	0.04	4.26	0.01	3.27	0.03	3.12	0.03
	$\beta_{"}$	post-level change	3.71	0.17	4.02	0.13	3.39	0.20	4.39	0.08	2.29	0.29	3.54	0.13
	$\beta_{\#}$	post-trend change	0.52	0.67	0.42	0.72	0.18	0.88	-1.54	0.19	0.12	0.91	0.21	0.83
	$\beta_{!} + \beta_{\#}$	post-trend	3.69	<.001	4.10	<.001	3.51	<.001	2.72	<.001	3.38	<.001	3.33	<.001

**Improvement in bathing**	$\beta_{!}$	pre-trend	1.91	0.02	2.24	0.004	2.07	0.01	1.72	0.10	2.17	0.01	1.62	0.01
	$\beta_{"}$	post-level change	1.10	0.31	2.00	0.05	1.62	0.12	-1.10	0.54	0.07	0.94	1.79	0.04
	$\beta_{\#}$	post-trend change	0.24	0.66	0.82	0.09	-0.08	0.85	0.31	0.74	0.02	0.97	0.32	0.35
	$\beta_{!} + \beta_{\#}$	post-trend	2.15	<.001	3.06	<.001	1.99	<.001	2.03	<.001	2.19	<.001	1.94	<.001

Notes: HHA is home health agency. Dual is dually eligible for Medicare and Medicaid. ADRD is Alzheimer's disease and related dementias. Hospitalization rate variables included both in-patient hospitalizations, readmissions, and outpatient observational visits. Timely initiation of care refers to home health spells which started within two days of an inpatient discharge. Other race included Asian, American Indian, Alaska native, native Hawaiian, or Pacific Islander. Parameters are estimated from an ITS regression model of $Y = \alpha + \beta_{!}T + \beta_{"}X + \beta_{\#}XT + \varepsilon$ where T = linear time and X = post-star ratings period. The pre-star ratings period was from January 2013 to December 2015 and the post-star ratings period was from January 2016 to December 2019. The unit is year.

eFigure 1. Study Flowchart

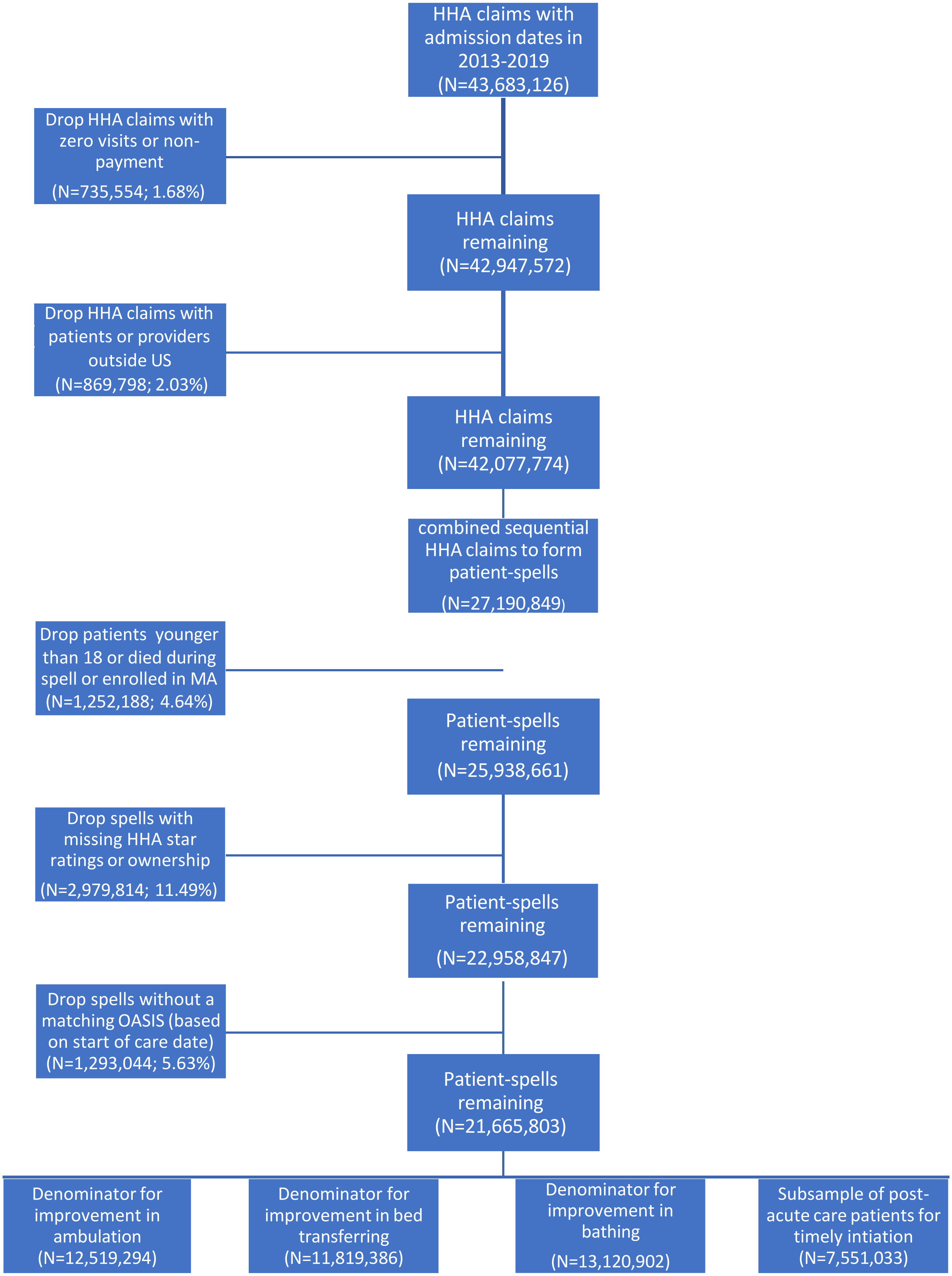

Notes: We started with all claims identified in the Medicare HHA FFS claims data (this included both HHA users who came from the community, hospital, and other post-acute care settings). We then excluded HHA claims with zero visits or non-payment and claims for patients who were under 18, had Medicare Advantage, resided outside of the U.S., or died during the spell. We also excluded HHA claims for patients with providers that were missing information about their quality of patient care star rating, patient satisfaction rating, or HHA ownership type. The denominator for each of the outcomes in the Interrupted Time Series analysis varied. The sample for the claims-based outcomes (e.g., hospitalization measures) was 21,655,803. The sample for the improvement in functional status measures varied based on the number of patient-spells which met the OASIS-based criteria for calculating an improvement in function measure (based on the OASIS start of care and discharge assessments). The sample for the timely initiation of care outcome was based on a subsample of HHA patients who were post-acute care HHA users (e.g., patient-spells that started within 14 days of inpatient discharge).

eFigure 2. Trends in claims-based outcomes by high quality HHA status

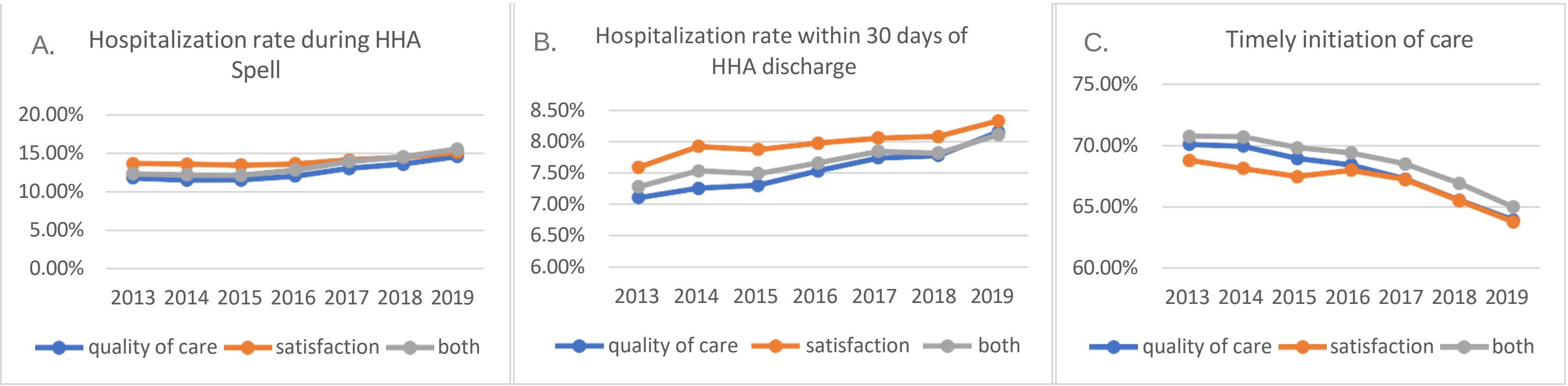

Notes: HHA is home health agency. Y-axis is the rate and x-axis is the year.

eFigure 3. Trends in OASIS-based outcomes by high quality HHA status

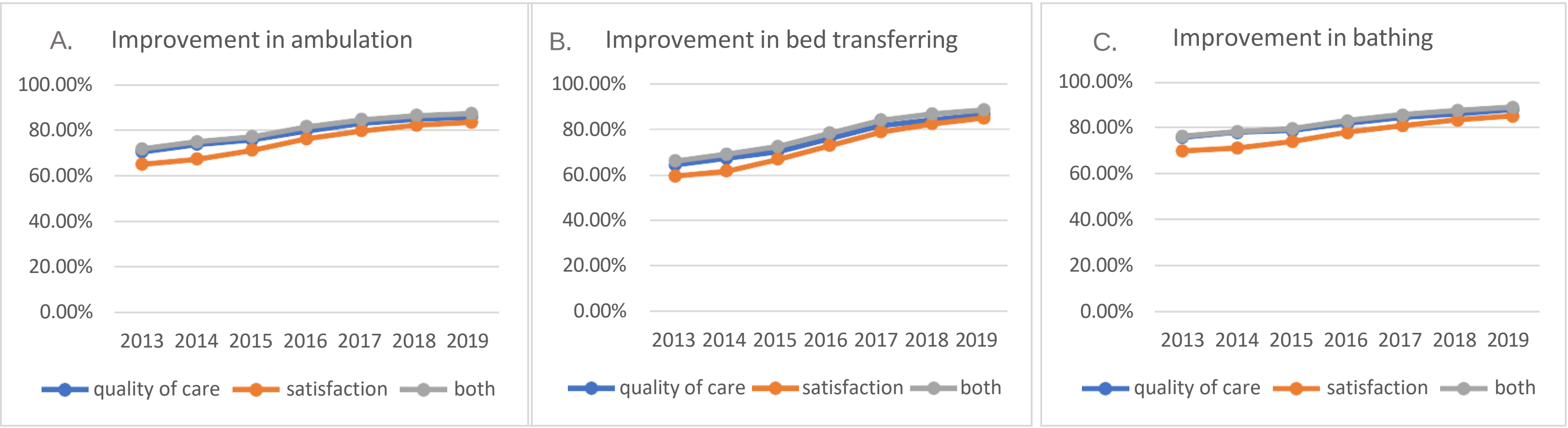

Notes: HHA is home health agency. Y-axis is the rate and x-axis is the year.
