# Supplementary material for: Claims-Based vs Agency-Reported Patient Outcomes Among Home Health Agencies, 2013-2019
Source: JAMA Netw Open. 2024 Apr 10;7(4):e245692. doi: 10.1001/jamanetworkopen.2024.5692 (PMC11007578; doi:10.1001/jamanetworkopen.2024.5692)
Supplement: Supplement 2. — Data Sharing Statement [file jamanetwopen-e245692-s002.pdf]

Data Sharing Statement

Chen. Claims-Based vs Agency-Reported Patient Outcomes Among Home Health Agencies, 2013-2019. *JAMA Netw Open*. Published April 10, 2024.
doi:10.1001/jamanetworkopen.2024.5692

Data
Data available: No

Additional Information
Explanation for why data not available: Our data will not be shared because it falls under a CMS data use agreement
